# Integrative analysis to select cancer candidate biomarkers to targeted validation

**DOI:** 10.18632/oncotarget.6018

**Published:** 2015-10-30

**Authors:** Rebeca Kawahara, Gabriela V. Meirelles, Henry Heberle, Romênia R. Domingues, Daniela C. Granato, Sami Yokoo, Rafael R. Canevarolo, Flavia V. Winck, Ana Carolina P. Ribeiro, Thaís Bianca Brandão, Paulo R. Filgueiras, Karen S. P. Cruz, José Alexandre Barbuto, Ronei J. Poppi, Rosane Minghim, Guilherme P. Telles, Felipe Paiva Fonseca, Jay W. Fox, Alan R. Santos-Silva, Ricardo D. Coletta, Nicholas E. Sherman, Adriana F. Paes Leme

**Affiliations:** ^1^ Laboratório de Espectrometria de Massas, Laboratório Nacional de Biociências, LNBio, CNPEM, Campinas, Brazil; ^2^ Instituto de Ciências Matemáticas e de Computação, Universidade de São Paulo, USP, São Carlos, Brazil; ^3^ Centro Infantil Boldrini, Campinas, Brazil; ^4^ Instituto do Câncer do Estado de São Paulo, Octavio Frias de Oliveira, São Paulo, Brazil; ^5^ Instituto de Química, Universidade Estadual de Campinas, UNICAMP, Piracicaba, Brazil; ^6^ Instituto de Ciências Biomédicas, Departamento de Imunologia, Universidade de São Paulo, USP, São Paulo, Brazil; ^7^ Instituto de Computação, Universidade Estadual de Campinas, UNICAMP, Campinas, Brazil; ^8^ Faculdade de Odontologia de Piracicaba, Universidade Estadual de Campinas, UNICAMP, Piracicaba, Brazil; ^9^ W. M. Keck Biomedical Mass Spectrometry Lab. University of Virginia, Charlottesville, Virginia, USA

**Keywords:** candidate biomarker, integrative analysis, proteomics, discovery, targeted

## Abstract

Targeted proteomics has flourished as the method of choice for prospecting for and validating potential candidate biomarkers in many diseases. However, challenges still remain due to the lack of standardized routines that can prioritize a limited number of proteins to be further validated in human samples. To help researchers identify candidate biomarkers that best characterize their samples under study, a well-designed integrative analysis pipeline, comprising MS-based discovery, feature selection methods, clustering techniques, bioinformatic analyses and targeted approaches was performed using discovery-based proteomic data from the secretomes of three classes of human cell lines (carcinoma, melanoma and non-cancerous). Three feature selection algorithms, namely, Beta-binomial, Nearest Shrunken Centroids (NSC), and Support Vector Machine-Recursive Features Elimination (SVM-RFE), indicated a panel of 137 candidate biomarkers for carcinoma and 271 for melanoma, which were differentially abundant between the tumor classes. We further tested the strength of the pipeline in selecting candidate biomarkers by immunoblotting, human tissue microarrays, label-free targeted MS and functional experiments. In conclusion, the proposed integrative analysis was able to pre-qualify and prioritize candidate biomarkers from discovery-based proteomics to targeted MS.

## INTRODUCTION

Discovery-based proteomics has been known as the most powerful tool for globally profiling proteomes and has been employed to mine biomarkers and therapeutic targets in many clinical conditions [1–5]. However, the contribution of novel molecules in clinical practice has been disappointing, and several reasons for failure have arisen in the long processes of biomarker and therapeutic target validation [6–8].

Recently, targeted proteomics has succeeded as the method of choice to overcome the drawbacks in validating and verifying potential biomarkers and therapeutic targets [7, 9–11]. Nevertheless, discovery-based proteomics can provide a large contribution in generating hypothesis-driven targets based on shotgun proteomics data [2, 12–15]. In addition to the bottleneck of discovery strategies such as the technical limitations of peptide quantification, undersampling, stochastic sampling process, and dynamic range [6, 8], there is a limited ability to use unbiased and robust methods to treat large-scale data as a whole when aiming to determine novel candidate biomarkers and therapeutic targets.

Ideally, for candidate biomarker outcomes in proteomics, the list of thousands of proteins identified by the discovery methods must be reduced into a smaller subset of features that will provide the maximal discriminating power between the conditions of optimal sensitivity and specificity. Many methods have already been proposed to compare the protein abundance in label-free shotgun proteomics with the aim of finding evidence for candidate biomarkers in proteomics datasets. Most of these methods are based on *p*-values that were derived from *t*-test [16, 17], analysis of variance (ANOVA) [18], Fisher's exact test [19, 20], etc. However, although these methods point to differences in protein abundance individually across conditions, they are limited in analyzing sets of data that contain multiple classes as well as providing an optimal feature set that capture the maximal variance in the data. In this work, we aimed to retrieve ranked lists of candidate biomarkers, which are considered here to be proteins that change in abundance on average between the different biological sample classes. A combination of three different methods was tested: a univariate method, Beta-binomial, a semi-multivariate method, Nearest Shrunken Centroids (NSC), and a multivariate method, Support Vector Machine-Recursive Features Elimination (SVM-RFE).

The mentioned methods were selected based on the following main reasons: (1) Beta-binomial is a univariate statistical method that was described by Pham et al. [21] to test the significance of differential protein abundances that were expressed in spectral counts in mass spectrometry-based proteomics. Moreover, experimental results from the same work showed that the Beta-binomial test performs favorably in comparison with other methods (e.g., Fisher's exact test, G-test, *t*-test and local-pooled-error technique) on several datasets in terms of both the true detection rate and the false positive rate and can also be applied in experiments with one or more replicates and in multiple condition comparisons; (2) NSC has already been shown to have the best performance compared to different univariate and multivariate methods in the previous work by Christin et al. [22]; (3) SVM-RFE is based on a machine-learning technique that has a completely different approach compared to NSC and was chosen as a complementary method to test both the results and the performances. NSC and SVM-RFE were combined to a double cross-validation step to define a final optimal set of discriminating proteins for distinguishing the three secretome classes with strictly low errors. Therefore, all of the three methods have already been separately tested and benchmarked for proteomics datasets, but they have not been used together in the same pipeline in which both the initial and final datasets were compared by different clustering techniques (heat map/hierarchical clustering and neighbor joining clustering) and silhouette coefficients. Furthermore, the final ranked lists of proteins were compared in a Venn diagram to be finally evaluated/validated by targeted proteomics in our proposed discovery-to-targeted pipeline.

In summary, the pipeline described in this work was tested on well-controlled data obtained from the secretomes of human melanoma (A2058 and SK-MEL-28), skin- and tongue-derived carcinoma (A431 and SCC-9, respectively) and non-cancerous (HaCaT and HEK293) cell lines. The MS-based discovery step was based on a routine shotgun analysis, which was followed by data analysis using the three mentioned approaches (Beta-binomial, NSC and SVM-RFE). These feature selection methods indicated that there was a panel of 137 proteins for carcinoma and 271 proteins for melanoma that were differentially abundant in these cell types. These selected proteins were then investigated by bioinformatics analyses, such as protein-protein interaction networks construction, enrichment analysis and literature curation. A protein network anticipated a potentially important role for the set of candidate biomarkers in the carcinoma, which was especially related to the complement and coagulation cascades, whereas in melanoma, the pathways associated with the cell cycle, cell adhesion and ubiquitin-mediated proteolysis were highlighted as being among the most altered in this pathologic condition. We further tested the strength of the pipeline in selecting candidate biomarkers by immunoblotting, human tissue microarrays, label-free targeted MS and functional experiments. It is noteworthy that the proteins Complement Factor B (CFB) and Complement C3 (C3) were found in significantly increased levels in oral squamous cell carcinoma (OSCC), compared to the adjacent normal tissue, and in human saliva from oral squamous cell carcinoma (OSCC) patients, using the pseudoSRM approach. Moreover, CFB knockdown decreased both the migration in the skin-derived epidermoid carcinoma (A431) cell line and chemotaxis in human macrophages. Furthermore, the pipeline was also applied to a published proteomics dataset of prostate cancer [23], and the results were compared with the approaches that were previously used.

In conclusion, we suggest that our proposed integrative analysis based on a discovery-to-targeted pipeline is especially valuable to better characterize candidate biomarkers for targeted MS verification.

## RESULTS

**Figure 1:**
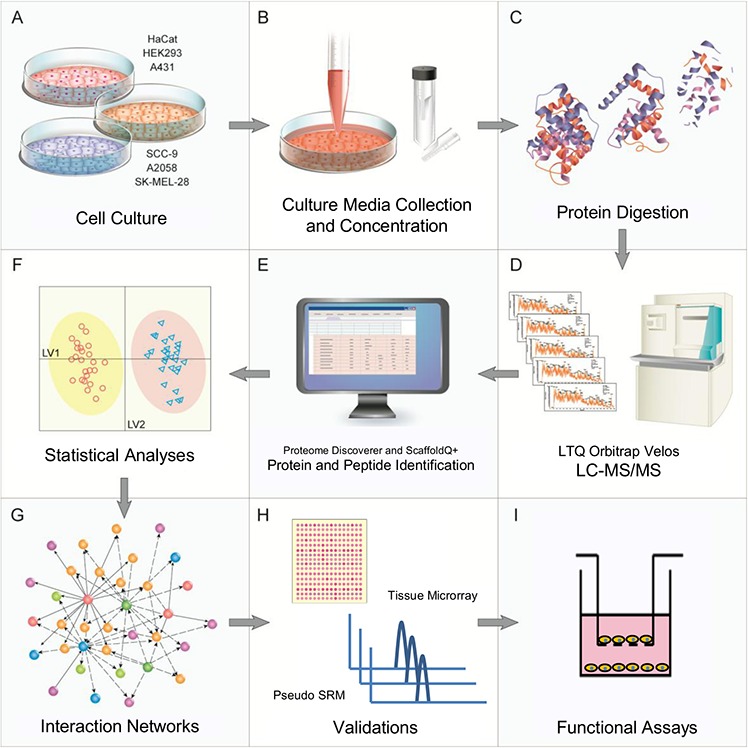
Experimental workflow and overview of the proteomics and bioinformatics analyses, validations and functional assays

A novel experimental pipeline has been proposed in this study to provide the bridge between discovery MS and targeted MS. This pipeline comprises four steps: MS-based discovery, feature selection analyses, bioinformatic tools to boost the extraction of biological information and targeted validation (Fig. 1). As a proof of concept, melanoma (A2058 and SK-MEL-28), skin and tongue-derived carcinoma (A431 and SCC-9, respectively) and non-cancerous cell lines (HaCaT and HEK293) had the protein content of their secretome collected, concentrated, trypsin digested and analyzed by LC-MS/MS. State-of-the-art univariate and multivariate methods were then employed to identify the most differentially abundant proteins among the three classes. A bioinformatics platform compiled these data into integrative networks that revealed cancer-specific biological information. These networks were able to characterize both carcinoma and melanoma cell archetypes and to point out pathways that could be potentially altered in each condition. Protein expression by tissue array in carcinoma and melanoma patients’ samples and by saliva samples, as well as gene silencing and functional experiments in cell lines provided validation for the proposed pipeline. Along with these findings, we also described the results that were obtained when the same pipeline was applied to an external dataset, which was a published study on prostate cancer [23]; these findings reinforced the effectiveness of our approach.

### Data analyses

#### Label-free quantitation in data dependent analysis

A list of 2,574 proteins with less than 1% FDR was generated by the Scaffold Q+ software from three biological replicates (Supplementary Table S1 and Table S2). Correlation analysis was performed to compare the output protein identification and quantitation (spectral counts) list of all possible pair-samples. High reproducibility was observed among the biological replicates, in which the R squared ranged from 0.64 to 0.96 (Supplementary Table S3).

From the 2,574 proteins, 877 proteins presented spectral counts ≤2 and were discarded from the following steps, leaving 1,697 remaining proteins for the subsequent analyses. The number of proteins that were identified in each experiment is shown in Table 1, whereas the number of proteins that were exclusive or shared by the cell lines is available in Supplementary Fig. S1.

**Table 1:** Number of proteins identified per experiment in each cell line

Cell Line	Number of identified Proteins by Mass Spectrometry		
	Exp.1	Exp.2	Exp.3
HaCaT	2015	2197	2201
HEK293	1690	1904	1861
A431	1879	1781	1884
SCC-9	2036	2213	2180
A2058	1950	1926	1974
SK-MEL-28	1660	1554	1770
Total		2574	
Total Spectra		151,221	

### Clustering and feature selection analyses of proteomics data

**Figure 2:**
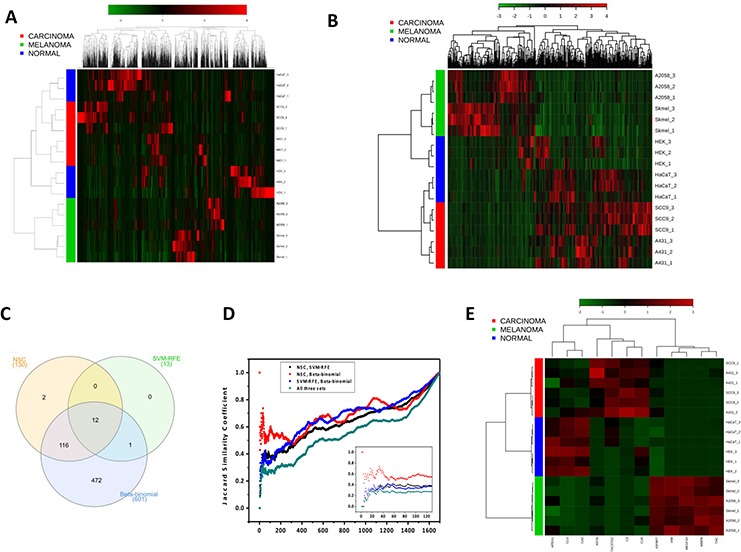
Comparison of the three feature selection methods (Beta-binomial, SVM-RFE and NSC) used to identify differentially abundant proteins among carcinoma, melanoma and non-cancerous cells **A.** Clustering of the whole secretome dataset before applying feature selection methods. From the 2,574 proteins identified and quantified by spectral counts, 1,697 (65.9%) compose the heat map. The 877 remaining proteins exhibited ≤2 spectral counts and were excluded from the analysis. **B.** Clustering after applying feature selection methods. 603 significant differentially abundant proteins among melanoma, carcinoma and non-cancerous classes selected by Beta-binomial, NSC and SVM-RFE analyses compose the heat map. **C.** Venn diagram showing the intersections among the optimal feature subsets (N) retrieved by the three methods. **D.** Jaccard similarity coefficient vs. the optimal feature subset (N) retrieved by each method. **E.** Clustering of the 12 significant differentially abundant proteins among melanoma, carcinoma and non-cancerous classes identified in the intersection of Beta-binomial, NSC and SVM-RFE analyses. The secretome dataset is composed by non-cancerous cells (HaCaT and HEK293), carcinoma (A-431 and SCC-9) and melanoma (A2038 and SK-MEL-28) cell lines.

An unsupervised hierarchical clustering performed with the 1,697 proteins mentioned above segregated the samples into two main classes, one that was composed exclusively by melanoma cell lines and the other that was composed by carcinoma and non-cancerous cells (Fig. 2A). Interestingly, the basal cluster segregated the cells according to their tissue of origin: from the epithelium-derived cell lines (SCC-9, A431 and HaCaT), from the skin-derived melanoma cells (SK-MEL-28 and A2058) and from the human kidney non-cancerous cells (HEK293), although a perfect group segregation for either non-cancerous or cancer cell lines was not observed. The result of this exploratory, unsupervised analysis indicated that melanoma's secretome is radically different from that produced by carcinoma and non-cancerous cells. That finding is probably due to the considerable similarities that are found between carcinoma and non-cancerous cell secretome, despite their obvious differences.

Aiming to evoke the most prominent dissimilarities among the groups, univariate, semi-multivariate and multivariate analyses were conducted, including the Beta-binomial, NSC and SVM-RFE methods, respectively. The models retrieved 601, 130 and 13 proteins, respectively, that were differentially abundant among the three secretome classes. These proteins were further associated with each class after a decision boundary step (Supplementary Table S4).

Both the SVM-RFE and NSC methods had their performance assessed in terms of double cross-validation errors, accuracy, sensitivity and specificity. These models presented 5.5% and 0% errors in double cross-validation, and 94.4% and 100% accuracy, respectively. Regarding the sensitivity and specificity, SVM-RFE showed 83.3% sensitivity for carcinoma and 100% for the other classes and 91.7% specificity for non-cancerous and 100% for the other classes, whereas NSC exhibited 100% sensitivity and specificity for all of the three classes (Supplementary Table S5).

The candidate biomarkers retrieved from the feature selection analyses were also used to perform the hierarchical clustering and heat maps again using the MetaboAnalyst platform (Fig. 2B). By this later analysis considering only the selected features, the same-class cell lines were clustered together, which confirms the set of retrieved candidate biomarkers as good discriminating proteins for distinguishing the three secretome classes (Fig. 2B). From this set, the Beta-binomial, NSC and SVM-RFE models retrieved 135, 32 and 4 characteristic proteins for carcinoma and 269, 78 and 6 proteins for melanoma, respectively (Supplementary Table S6).

Furthermore, the final ranked lists of 601, 130 and 13 candidate biomarkers for all three classes, which resulted from the Beta-binomial, NSC and SVM-RFE models, respectively, were compared by a Venn diagram and by the Jaccard similarity coefficient. This comparison showed that the SVM-RFE optimal feature subset is almost completely shared by the NSC and Beta-binomial models (12 out of 13 proteins) and that the NSC optimal feature subset is almost completely shared by the Beta-binomial model (128 out of 130 proteins) (Fig. 2C). Moreover, based on the Jaccard similarity coefficient, the comparison of protein rankings resulting from the three models (Beta-binomial, NSC and SVM-RFE) is almost linear, not showing large variances in the similarity coefficient from the 10th to the 130th position in the ranking (green line, inset of Fig. 2D). This means that the three models have almost a constant similarity coefficient (~0.3) from the 10th to the 130th position in the ranking. From the 130th to the ~200th position there is an increase in the slope of the curve reflecting an increase in the similarity coefficient (Fig. 2D, main graphic). Notably, the SVM-RFE model was able to discriminate the three classes based on the smallest set of only 13 proteins (gene names: C3, CLU, MEGF10, MMP8, BANF1, VIM, APEX1, CA2, TACSTD2, KRT8, TNC, C1R and IGFBP7), of which only BANF1 was not retrieved by the other two methods. In contrast, as expected for a univariate method, the Beta-binomial model yielded the largest set of differentially abundant proteins, covering all of the proteins that were retrieved by the two multivariate methods (except for two proteins from NSC). Notably, using only the 12 candidate biomarkers retrieved by the three feature selection methods, a perfect segregation among the carcinoma, melanoma and non-cancerous classes was also observed (Fig. 2E). The complete ranked protein lists that resulted from the three methods are available in Supplementary Table S4; the plots showing the spectral count distribution in the melanoma, carcinoma and non-cancerous cells for the 130 candidate biomarkers retrieved from NSC can be found in Supplementary Fig. S2.

**Figure 3:**
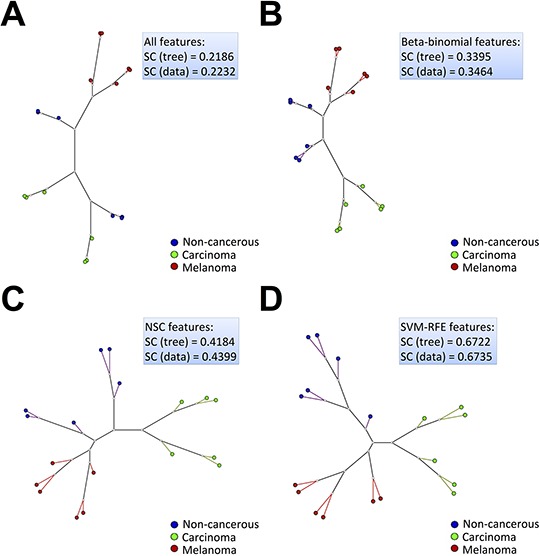
Neighbor joining (NJ) clustering calculated from a Euclidean distance matrix of the secretome dataset samples, considering **A.** all features (1,697 proteins), **B.** Beta-binomial (601 proteins), **C.** NSC (130 proteins) and **D.** SVM-RFE (13 proteins) features. SC (tree) stands for silhouette coefficient calculated from the NJ tree and SC (data) stands for silhouette coefficient calculated directly from the original data of each analysis.

In addition to the hierarchical clustering and heat map analysis, similarity trees were constructed from a Euclidean distance matrix of the 18 samples considered for the feature selection analyses. Fig. 3 shows that the Neighbor Joining (NJ) trees were capable of showing the most similar elements of the set, which were present in the same or in nearby branches. In this work, a reasonable separation of the three classes was found when the whole dataset was considered in the NJ tree construction (Fig. 3A) (silhouette coefficient, SC > 0.2). However, as was also shown by the previous unsupervised hierarchical clustering and heat map analysis for the whole dataset, the melanoma samples were the only ones that clustered together in the same or nearby branches connected to the same node, separated from the carcinoma and non-cancerous samples, which were distributed in different branches and did not show a perfect segregation in their respective classes. On the other hand, as expected, there was an improvement in the NJ clustering and silhouette coefficients that were calculated after feature selection, considering only the candidate biomarkers that were retrieved from each model (Fig. 3B–3D). For instance, if a labeled dataset has a silhouette coefficient that is closer to 1 (ranging from −1 to 1), then the classes are almost homogeneous and different from each other and classifiers will probably perform well in constructing a good model with a low double cross-validation error. Consequently, this finding also means that sets of good discriminating features (proteins) among the classes could be retrieved by feature selection analysis.

Besides the feature selection methods described above, the univariate ANOVA test was also performed in our data to compare our results to a classical statistical method. In total, ANOVA retrieved 875 differentially abundant proteins (*p* < 0.05, Supplementary Table S4). The ANOVA result corroborates the results obtained by the three methods proposed in our pipeline, which can be observed by the intersections in a second Venn diagram built for the four output lists of candidate biomarkers (Supplementary Fig. S3). However, it brought over 384 exclusive proteins from a total of 987 proteins selected by the four methods (~40%), which is a large percentage for ANOVA to be considered in the pipeline as a method that could contribute with an optimal set of features for selecting candidates. Moreover, when we compare the rank index of candidate biomarkers retrieved by the three feature selection methods proposed in the pipeline to the rank index given by ANOVA, we observed that the reduced list of candidates selected by the three approaches were not the top candidates chosen by ANOVA (Supplementary Table S4).

The same feature selection analyses were also performed for a published proteomics dataset on prostate cancer [23] to validate our approach. The output final ranked lists of candidate biomarkers that resulted from each method (Supplementary Tables S7–S11) were analyzed by a Venn diagram, which showed that five candidates that were validated/verified by different approaches in the original work by Kim et al. [23] were also identified in the intersections of the Venn diagram (Supplementary Table S10 and Supplementary Fig. S4), which reinforces the effectiveness of the proposed discovery-to-targeted pipeline.

### Bioinformatics analyses

**Figure 4:**
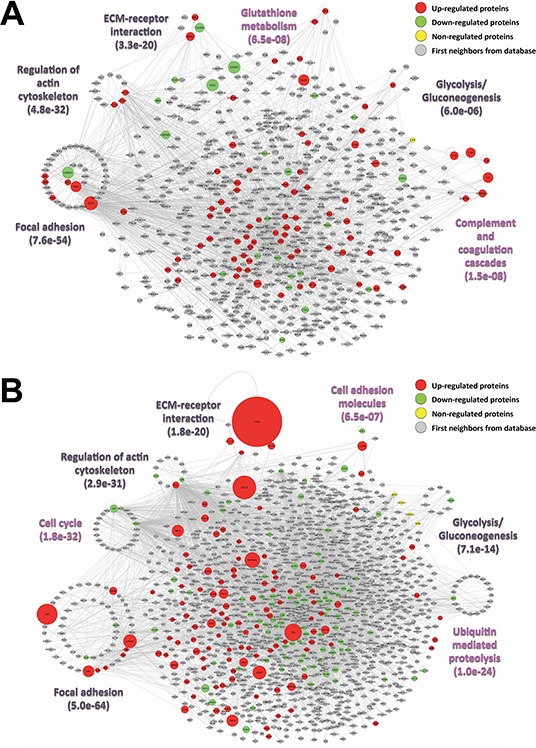
Interaction networks of the identified **A.** carcinoma and **B.** melanoma candidate biomarkers by Beta-binomial, NSC and SVM-RFE analyses. The selected most relevant enriched KEGG pathways (*p* ≤ 0.05) among the up-regulated (red), down-regulated (green), non-regulated (yellow) and background intermediary proteins (grey) from the IIS database are depicted by clustering with a circular layout proteins involved in each respective pathway. Clusters were assigned only to pathways containing more than three proteins with at least one protein from the proteome dataset (disease pathways or pathways specific for defined cell types were not considered); proteins belonging to more than one pathway were assigned to the pathway clusters with the best enrichment *p*-values; some proteins were also assigned to different pathway clusters based on complementary data from the Uniprot database. In magenta, pathway clusters exclusive of each network; in black, pathway clusters in common. The node sizes of up, down and non-regulated proteins are proportional to their fold change (−1.3 ≥ fold change ≥1.3, compared to the non-cancerous class). The protein-protein networks were built using the IIS software and visualized using Cytoscape.

To evaluate the protein interaction profile within each tumor class, protein-protein interaction networks were constructed using the IIS software for the candidate biomarkers obtained by the feature selection methods and estimated to be associated to carcinoma or melanoma classes (Supplementary Table S6.). The networks represent a “snapshot” of the secretome of both classes, which illustrate the proteins that most probably play a role in the secretome regulation of each tumor type (Fig. 4, Supplementary Tables S12 and S13). Our network analysis showed direct connections between the identified candidate biomarkers and the proteins that are involved in enriched KEGG pathways (*p* ≤ 0.05), highlighting the most important pathways that are likely to be activated/inhibited in each disease. Accordingly, the networks suggested a potentially important role for carcinoma biomarkers in focal adhesion, regulation of actin cytoskeleton, ECM-receptor interaction, glutathione metabolism, glycolysis/gluconeogenesis and, especially, complement and coagulation cascades, which were not enriched among the melanoma biomarkers (Fig. 4A). In contrast, focal adhesion, cell cycle, regulation of actin cytoskeleton, ECM-receptor interaction, cell adhesion molecules, glycolysis/gluconeogenesis and ubiquitin-mediated proteolysis were identified as being significantly enriched (*p* ≤ 0.05) pathways in the melanoma secretome; these pathways presented at least one candidate biomarker that participates in each of them. Focal adhesion and ECM-receptor interaction, especially, appear to have a relevant role in melanoma due to the outstanding, differential abundance of the proteins that belong to these two pathways (Fig. 4B). All of the significant enriched pathways that were extracted from both carcinoma and melanoma networks are listed in Supplementary Tables S12 and S13, respectively.

Furthermore, to verify whether the candidate biomarkers for carcinoma and melanoma had been previously described as related to cancer or to some biomarker application, an Ingenuity (IPA) biomarker filter module analysis and a search in the Human Protein Atlas Database were performed.

The IPA biomarker analysis retrieved 45 (32%) proteins of the candidate biomarkers identified in our study for carcinoma and 76 (28%) for melanoma, which have been previously described as being strongly associated with cancer and/or involved in biomarker applications. Likewise, the Human Protein Atlas Database retrieved 32 (23%) and 60 (22%) proteins of the carcinoma and melanoma candidates, respectively, which were found to be previously associated with cancer (Supplementary Table S6).

These analyses were also performed for the set of candidate biomarkers that were retrieved by the feature selection methods applied to the prostate cancer proteomics dataset published by Kim et al. [23]. Interestingly, from the 47 proteins that were identified in the intersection of the three methods (Supplementary Table S10 and Supplementary Fig. S4), IPA determined that 13 (28%) proteins had been associated with some biomarker application, whereas 17 (36%) proteins had already been described as candidate cancer biomarkers according to the Human Protein Atlas Database (Supplementary Table S11).

### Validation of the expression of candidate markers for melanoma and carcinoma

Based on the available commercial antibodies, six up-regulated proteins retrieved by Beta-binomial, NSC and/or SVM-RFE models were chosen to be validated by immunoblotting. The overexpression of Fibronectin (FN1), Tenascin-C (TNC) and Growth/differentiation factor 15 (GDF15) in melanoma cell lines and of Complement factor B (CFB), Talin-1 (TLN1) and Epidermal growth factor receptor (EGFR) in carcinoma cell lines was confirmed in the conditioned media of the six cell lines (Supplementary Fig. S5).

**Figure 5:**
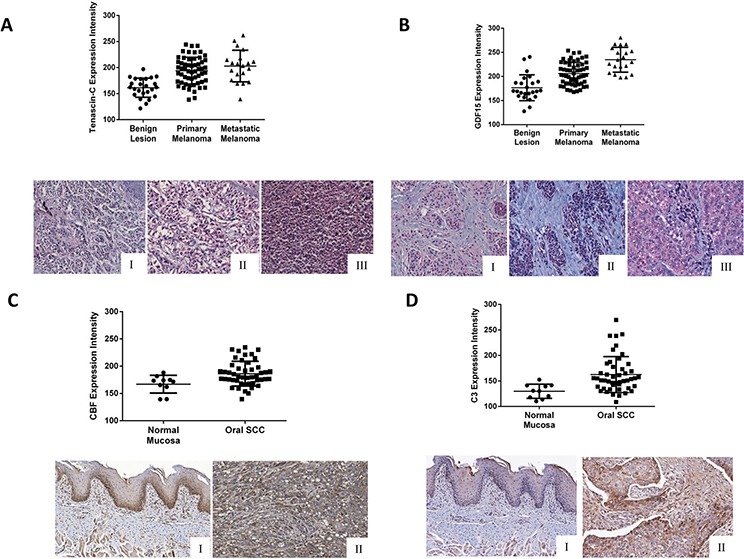
Validation of the higher expression of **A.** tenascin-C and **B.** GDF15 (I-Benign lesion; II- Primary Melanoma; III-Metastatic Melanoma) on melanoma cancer tissue microarrays and **C.** CFB and **D.** C3 (I- Normal Mucosa; II- Oral SCC) on carcinoma cancer tissue microarrays. Tenascin-C showed statistically significant expression among the categories benign lesion, primary melanoma and metastatic melanoma, but not between primary melanoma and metastatic melanoma (One-way ANOVA, benign lesion vs. primary melanoma, *p* < 0.0001; benign lesion vs. metastatic melanoma, *p* < 0.0009; primary melanoma vs. metastatic melanoma, *p* = 0.1748). GDF15 showed statistically significant expression among the categories benign lesion, primary melanoma and metastatic melanoma (One-way ANOVA, benign lesion vs. primary melanoma, *p* < 0.0001; benign lesion vs. metastatic melanoma, *p* < 0.0001; primary melanoma vs. metastatic melanoma, *p* < 0.0001). CFB and C3 showed higher expression in OSCC compared with normal mucosa (Mann Whitney U, *p* = 0.009 and *p* = 0.0005, respectively).

To further investigate whether those candidate markers were clinically associated with tumors, we used tissue microarrays with human melanoma samples to examine TNC and GDF15 expression. Both TNC and GDF15 were found in the cytoplasm of the nevoid cells, with significantly higher expression levels in the tumor cells compared with normal cells (Fig. 5A and 5B). Interestingly, the expression of GDF15 was significantly higher in metastatic than in primary melanomas (one-way ANOVA, *p* < 0.0001).

The expression of CFB was limited to the cytoplasm of the basal and suprabasal layers of the normal oral tissue, whereas broad positivity was found in the tumor cells (Fig. 5C). Considering the intensity levels, the expression of CFB was significantly higher in tumors compared with normal mucosa (Mann Whitney U test, *p* = 0.0057, Fig. 5C). Similarly, C3 was found in the cytoplasm of the epithelial cells, but the intensity was significantly higher in tumor cells compared to normal keratinocytes (Mann Whitney U test, *p* = 0.016, Fig. 5D). Immunoreactivity for C3 was also observed in inflammatory and endothelial cells.

### Label-free targeted MS

To further test the strength of the pipeline in selecting candidate biomarkers that were retrieved by all of the methods, we prioritized two candidates from the carcinoma secretome to have their abundance assessed in the saliva of Oral Squamous Cell Carcinoma (OSCC) patients, as a first step toward biomarker evaluation in clinical samples. We believe that saliva is a promising biofluid for investigation due to the ease of its collection and its direct contact with oral cancer lesions. The samples were collected from OSCC patients, who were divided into two groups: patients who had undergone surgical resection (named as “no lesion”, *n* = 7) and those who had active oral malignant lesion (named as “lesion”, *n* = 10) at the time of the saliva collection (Supplementary Table S14). Saliva samples from healthy individuals were also used as a control (*n* = 7).

We validated both C3 and CFB, and C3 was selected for being top ranked in the three feature selection analysis results (SVM-RFE rank index = 1; NSC rank index = 13; Beta-binomial rank index = 15) as well as for being assigned to the complement and coagulation cascades pathway, an enriched (*p*-value = 1.52e−08) carcinoma-exclusive KEGG pathway that is based on complementary data from the Uniprot database. Regarding CFB, it was simultaneously retrieved by NSC (rank index = 33) and Beta-binomial (rank index = 31), and very importantly, it takes part in the same pathway as C3. In addition, both CFB and C3 have not been previously reported to be related to cancer biomarkers, according to the IPA biomarker analysis and The Human Protein Atlas.

We selected two peptides for each protein based on the following criteria: uniqueness, high relative abundance, MS/MS spectral quality, experimental observation of proteomic data repositories (PeptideAtlas) and DDA analysis performed using LTQ Orbitrap Velos. The targeted proteomics were performed using selected ion monitoring (SIM) of each targeted peptide in high mass resolution for quantitation, followed by scheduled MS/MS for confirming targeted peptide sequences (Supplementary Table S15). The peak area of each targeted peptide was extracted using the Xcalibur software (Supplementary Table S16) and normalized to the angiotensin internal standard (Supplementary Table S17), spiked in all of the samples to a final concentration of 5 fmol/μl to correct run-to-run variations.

**Figure 6:**
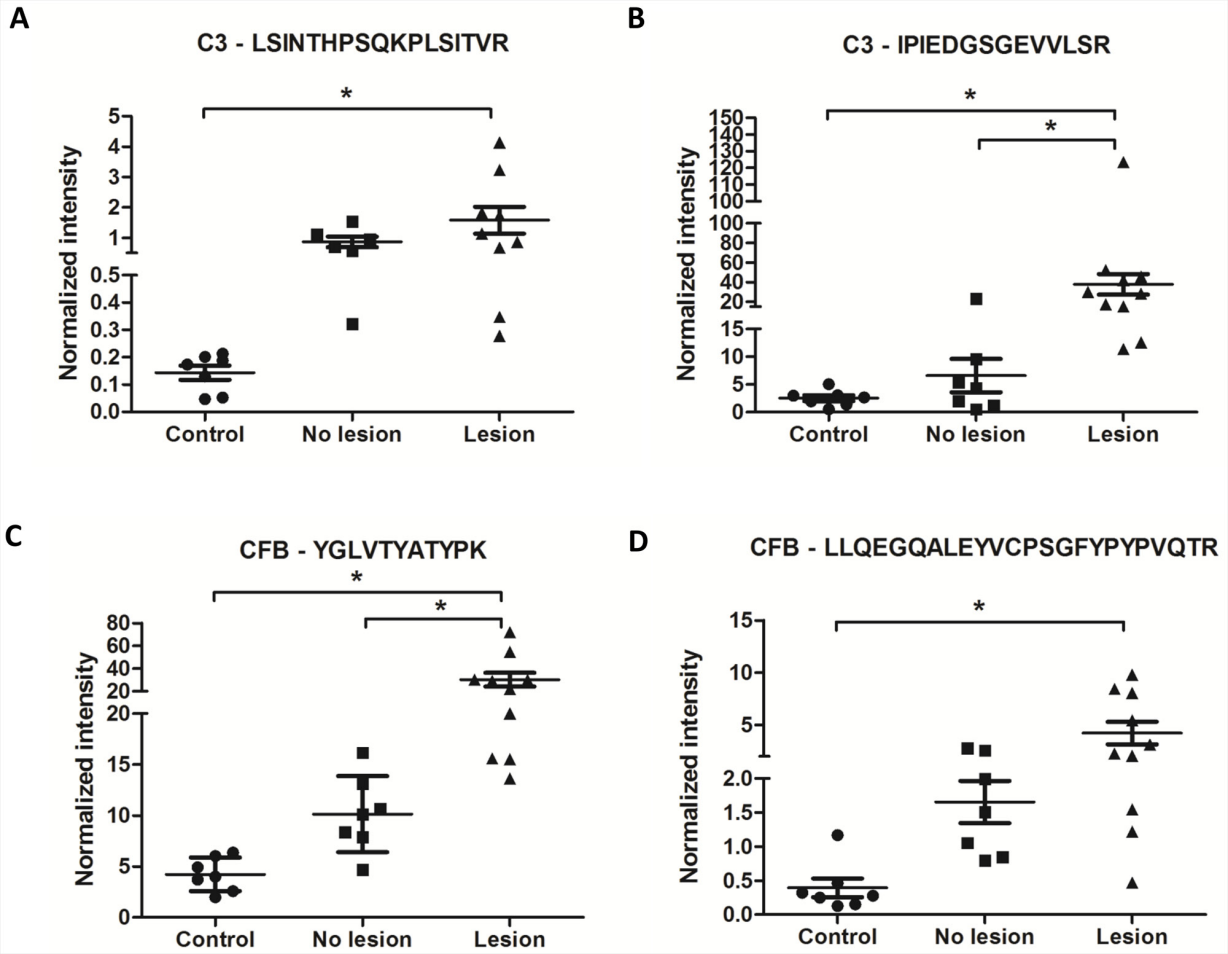
CFB and C3 peptides showed higher normalized intensities in OSCC saliva samples than in healthy saliva samples PseudoSRM analytical approach for peptides of C3 (precursor m/z 631.05, +3; 735.89, +2) and CFB (precursor m/z 638.33, +2; 939.13, +3) normalized with 5 fmol/μl of angiotensin (m/z 432.89, +3) as an internal reference peptide. These data represent two technical replicates of saliva samples from healthy patients (*n* = 7), saliva samples from patients who undergone surgical resection of OSCC (named no lesion, *n* = 7) and saliva samples from patients with active OSCC lesion without any treatment (named lesion, *n* = 10) (ANOVA followed by Tukey's test). The normalization to the internal reference peptide was performed for each run.

The averages of the normalized intensities of each peptide in each sample were visualized in a scatter plot graph, and ANOVA followed by Tukey's test was performed to evaluate the statistical significance among the conditions (Fig. 6). It was observed that the saliva from the OSCC patients with lesions had a significantly higher normalized intensity of the precursor area of both CFB and C3 compared to healthy subjects with respect to all of the peptides evaluated (Fig. 6A–6D). Additionally, the C3 peptide, IPIEDGSGEVVLSR, and the CFB peptide, YGLVTYATYPK, both showed a significant difference between the patients without a lesion and with a lesion (Fig. 6B and 6C). When the sum of the three transitions of each peptide (normalized by the sum of the three transitions of the angiotensin internal peptide) was considered, similar results were found (Supplementary Fig. S6). The extracted ion current peak area from MS1 and the three MS/MS transitions as well as the CV% of each replicate are shown in Supplementary Tables S16 and S17.

The performance of the method was evaluated using angiotensin spiked in the HEK cell lysate digest (500 ng) in five different concentrations, for which each sample was run in triplicate. Good linearity (*R* = 0.998) (Supplementary Fig. S7) and CV <15% were observed at three concentration points (Supplementary Table S18).

### CFB knockdown decreased the migration of A431 cells and impaired the chemoattraction of human macrophages

The final approach that was used to explore the strength of the pipeline was to perform functional assays, which was chosen because of the implication that CFB could have in biological processes that are related to cancer.

**Figure 7:**
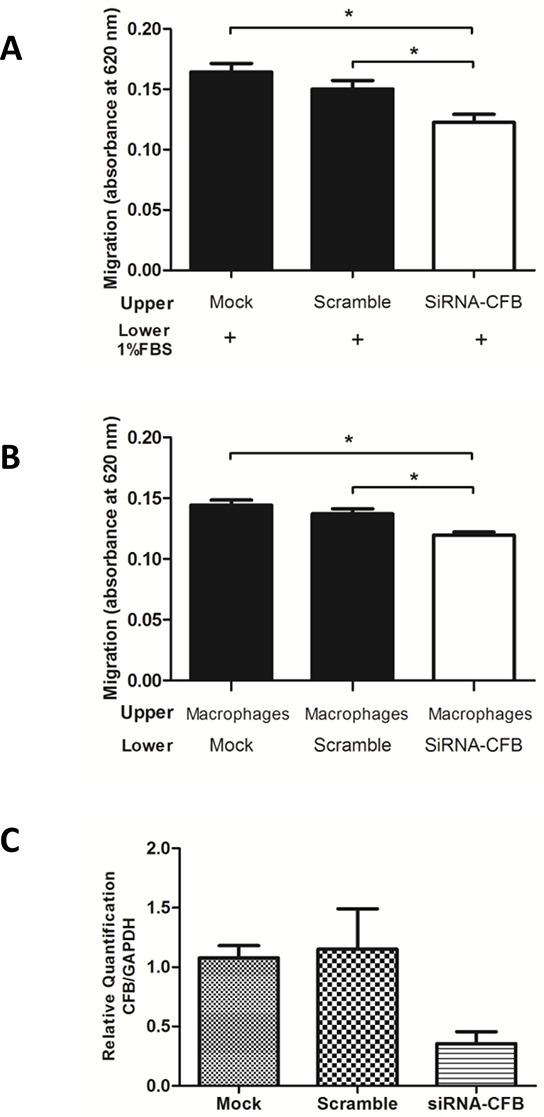
CFB knockdown decreased the migration of skin-derived epidermoid carcinoma (A431) cells and reduced the chemotaxis of human macrophages **A.** A431/untreated (mock), A431/control (scrambled) and A431/siRNA CFB cells were seeded in serum-free media in the upper chamber of a 96-well transwell plates. RPMI media, which was supplemented with 1% FBS, was added in the lower chamber (*n* = 2, triplicate, one-way ANOVA followed by Tukey's test, *p < 0.05). **B.** Chemotaxis of human macrophages was reduced when were seeded in the upper chamber, and A431 cells treated with mock, control siRNA and siRNA against CFB were added in the lower chamber of the transwell (*n* = 2, triplicate, a one-way ANOVA followed by Tukey's test, *p < 0.05). C. Real-time quantitative PCR confirms the expression of CFB after transient transfections in A431 cells. The data were normalized with the (glyceraldehyde-3-phosphate dehydrogenase gene was used as internal reference). Each bar represents mean ± SD of three independent experiments.

It is well known that complement proteins are considered to be powerful proinflammatory molecules in the body [24], and recently, C3 was evidenced as a key player in the production and activation of ovarian cancer growth and progression [25]; however, there is still no evidence associated with CFB in oral tumorigenic processes. Therefore, we performed the knockdown of CFB in the A431 cell line using siRNA, and we first evaluated the effect of this protein in cell migration. As observed in Fig. 7A, CFB knockdown decreased the migration of A431 cells compared with mock and control siRNAs (one-way ANOVA followed by Tukey's test, *n* = 2, *p* < 0.001).

Furthermore, CFB is a protein that is secreted by macrophages, fibroblasts, endothelial cells and tumor cells [24]. Therefore, we evaluated the paracrine effect that CFB depletion in tumor cells could exert on macrophage chemotaxis. To accomplish this goal, macrophages were placed in the upper chamber of a transwell plate, whereas A431 cells that were treated either with mock, control siRNA or siRNA against CFB were laid in the lower chamber of the same plate. Macrophage migration through the transwell was significantly reduced in CFB knockdown A431 cells, which suggested that the presence of this protein in the conditioned medium had the ability to modulate macrophage taxis (Fig. 7B, one-way ANOVA followed by Tukey's test, *n* = 2, *p* < 0.001). Cell knockdown for CFB was confirmed by qRT-PCR (Fig. 7C). Together, these experiments showed that CFB protein plays a role in tumorigenic processes such as macrophage chemotaxis and cell migration.

## DISCUSSION

This study introduced an integrative analysis based on a pipeline that combines MS-based discovery followed by feature selection methods, clustering, Venn diagram, network analyses, and targeted approaches to generate reliable hypothesis-driven targets based on shotgun proteomics, to provide a bridge between discovery MS and targeted MS.

Well-controlled proteomic data from the secretomes of three classes of human cell lines were analyzed with respect to the protein content of their secretomes using discovery-based proteomics. To retrieve ranked lists of candidate biomarkers, a combination of a univariate method (Beta-binomial), a semi-multivariate method (NSC) and a multivariate method (SVM-RFE) was tested. The great advantage of the feature selection methods used in this work is that NSC and SVM-RFE models summarize thousands of features into a few key components that capture the maximal variance in the data. Together with the Beta-binomial model, which was used to test the significance of differential protein abundances expressed in spectral counts, the three ranked lists of candidate biomarkers were retrieved and compared using the Jaccard similarity coefficient and a Venn diagram, to be further evaluated by bioinformatic analyses (interaction networks, pathway enrichment and biomarker investigation) and targeted proteomics. Moreover, both the initial and final datasets were compared by different clustering techniques (heat map/hierarchical clustering and neighbor joining clustering) and silhouette coefficients, which showed an improvement in both the clustering and silhouettes after feature selection and served as a proof-of-concept that the set of retrieved candidates was constituted by good discriminating proteins for distinguishing the three secretome classes.

Our approach proved to be of great value in tracking potentially promising candidate biomarkers from proteomics data, since many of these proteins have already been demonstrated to be associated with cancer. For example, both the IPA and Human Protein Atlas Database analyses retrieved, respectively, 32% and 23% of the carcinoma candidates and 28% and 22% of the melanoma candidates that were previously found to be associated with cancer (Supplementary Table S6).

To further explore the biological role of these findings, we integrated the proteomics data into networks that highlighted the direct connections between the selected candidates and their possible roles in each disease (Fig. 4). Despite the highly complex and dynamic nature of network biology [26], our interaction networks enabled us to easily sum up all of the proteomics data and decipher the main cellular contexts of the candidate biomarkers in carcinomas and melanomas. The proteins were clustered in highly enriched pathways and were visualized by their relative abundances through node colors and sizes; most of the candidates were found to be related to cell-cell communication and interactions. Specifically, this analysis retrieved exclusive pathways for the carcinoma candidates, such as complement and coagulation cascades (Fig. 4A), and for the melanoma candidates, such as cellular functions associated with the cell cycle, cell adhesion and ubiquitin-mediated proteolysis (Fig. 4B).

In the final steps of the proposed pipeline, we tested the promising proteins CFB and C3 as candidate carcinoma biomarkers, which in addition to being associated with the enriched complement and coagulation cascade pathway, were validated using immunoblotting, tissue microarrays and retrieved in the intersections of the feature selection methods.

In the first approach, we have indeed found a higher expression of CFB and C3 proteins using a label-free pseudoSRM analysis of human saliva from Oral Squamous Cell Carcinoma (OSCC) patients in comparison with healthy individuals (Fig. 6). Because saliva is simple to collect and process, it may lead to a useful clinical tool for the noninvasive prognosis of oral cancer in the future [14, 27]. It is important to highlight that oral cancer, primarily OSCC, is the sixth most common cancer and is an important public health concern worldwide [28], with low 5-year survival rate due to the compounding factors of late detection and lack of truly effective therapies [29, 30].

Although complement components are primarily synthesized locally by many cell types, including macrophages, fibroblasts and endothelial cells [31], some neoplastic cells have also been shown to synthesize and secrete components of the C system [32–34]; however, the role of the complement system in tumor cells remains controversial. Recently, an autocrine effect of complement proteins has been shown; specifically, C3 and C5 are secreted by ovarian cancer cells on tumor growth [25]. It is also well known that the complement system contributes to inflammation, mainly through C3a and C5a, which are the most powerful proinflammatory anaphylatoxins in the body [24, 35] and to immunosuppression through components such as C3, C4 and C5a [36]. Interestingly, the adopting characteristics that involve the inflammatory state and the ability to avoid the immune system have been emerging as hallmarks in cancer [37].

Because no evidence was shown regarding the function of CFB in cancer cells, in the second approach, we explored the functional role of CFB in tumorigenic processes, such as cell migration and chemotaxis. The CFB knockdown in the skin-derived epidermoid carcinoma (A431) cells decreased the ability of the cells to migrate and the chemotaxis of human macrophages (Fig. 7A–7B), which suggests that, in addition to a higher expression in OSCC tissues and saliva, CFB might mediate these events in carcinomas.

Furthermore, we applied our pipeline for a published label-free proteomic dataset [23], which previously reported the identification of 133 significantly differentially expressed proteins in extracapsular and organ-confined prostate cancer direct-EPS fluids using a hierarchical Bayesian statistical algorithm known as QSpec. Among these proteins, five proteins were validated/verified using different methods (ELISA, Western blot and SRM-MS). Using the feature selection methods proposed in our pipeline, the same five proteins validated by Kim et al. [23] were also found in the intersections of our Venn diagram analysis (SFN, MME, TGM4, TIMP1 and PARK7, Supplementary Table S10), reinforcing the effectiveness of our approach.

In conclusion, the proposed integrative analysis based on a discovery-to-targeted pipeline was able to pre-qualify potential candidates from discovery-based proteomics to targeted MS and can contribute to the next phases of biomarker development in translational initiatives to drive either patient stratification, decision making or intervention.

## MATERIALS AND METHODS

### Cell culture

SCC-9 cells (squamous cell carcinoma, a tumor cell line originated from a human tongue squamous cell carcinoma) were obtained from the American Type Culture Collection (ATCC, Manassas, VA) and cultured in DMEM/Ham's F12 medium (Cultilab), supplemented with 10% fetal bovine serum (FBS), antibiotics and 0.4 μg/ml hydrocortisone. Human keratinocyte HaCaT (immortalized, but not transformed, epithelial cell line), Human embryonic kidney HEK293 and human melanoma A2058 cell lines (isolated from a metastatic site in a skin-derived lymph node) were maintained in DMEM containing 10% FBS and antibiotics. Human melanoma SK-MEL-28 cells (malignant skin-derived melanoma cell line) and human epidermoid carcinoma A431 (skin-derived epidermoid carcinoma cell line) were grown in Roswell Park Memorial Institute (RPMI) − 1640 medium supplemented with 10% FBS and antibiotics. All cells were maintained at 37°C in a 5% CO_2_ atmosphere.

### Sample preparation for MS

*Label-free Discovery Proteomics*: Cells at 80% confluence (two 15-cm dishes per condition per experiment) were gently washed three times in phosphate buffered saline (PBS) and incubated in a serum-free medium (20 ml per dish) for 24 h at 37°C. After collection of the conditioned media EDTA and PMSF (Phenylmethylsulfonyl fluoride) were added at a final concentration of 1 mM. Cell debris and intact cells were eliminated by centrifugation at 4,000 rpm (Eppendorf Centrifuge 5810R) for 5 min at 4°C and the conditioned media were subsequently concentrated using a 3000-Dalton centrifugal filter (Millipore, Billerica, MA) at 4,000 x *g* at 4°C. Protein concentrations were determined using a Bradford assay (Bio-Rad, Hercules, CA, USA). Proteins (80 μg) were treated with a final concentration of 1.6 M urea, following reduction (5 mM dithiothreitol, 25 min at 56°C), alkylation (14 mM iodoacetamide, 30 min at room temperature in the dark) and digestion with trypsin (1:50, w/w). The reaction was stopped with 1% TFA and desalted with Sep-pack cartridges (Waters). The samples were dried in a vacuum concentrator, reconstituted in 0.1% formic acid and analyzed by LC-MS/MS. Three independent experiments were performed for each cell line.

*Label-free Targeted Proteomics*: The saliva was collected from healthy individuals (*n* = 7), patients who underwent surgical resection (named as no lesion, *n* = 7) and patients with active oral malignant lesion (named as lesion, *n* = 10). Individuals were asked to first rinse their mouth with 5 ml of drinking water and to harvest the saliva into a glass receptacle. Saliva was then aliquot in 2 ml tubes and immediately frozen at −80°C. All patients and volunteers enrolled signed a formulary stating their awareness and consent for the study, approved by the Research Ethics Committee of Faculdade de Odontologia de Piracicaba, Universidade Estadual de Campinas, UNICAMP, Piracicaba, Brazil.

Proteins were extracted by homogenizing the 100 μl of whole saliva with 100 μl of a solution containing 100 mM Tris-HCl, pH 7.5, 8 M urea, 2 M thiourea containing Protease Inhibitor Cocktail cOmplete Mini Tablets (Roche, Auckland New Zealand), 5 mM EDTA, 1 mM PMSF and 1 mM DTT. Samples were sonicated for 10 min and centrifuged at 10,000 x *g* for 5 min. Protein concentrations were determined using a Bradford assay (Bio-Rad, Hercules, CA, USA). Five fmol/μl of angiotensin synthetic peptide (precursor m/z 432.8998, +3, DRVYIHPFHL, Sigma-Aldrich) were added to each peptide mixture (600 ng of total protein) as an internal reference peptide.

### Mass spectrometric analysis

Peptide samples were analyzed on an ETD-enabled LTQ Orbitrap Velos Mass Spectrometer (Thermo Fisher Scientific) connected to a nanoflow liquid chromatography column (LC-MS/MS) by an EASY-nLC System (Proxeon Biosystem) through a Proxeon nanoelectrospray ion source. The mass spectrometry analysis for label free discovery and target proteomics as well as the proteomics data analysis for protein identification are described in the Supplementary Material and Methods.

### Feature selection analyses of proteomics data

#### Heat map and hierarchical clustering analyses

Files containing the identified proteins and their spectral counts were used for the clustering and heat maps generation, as well as to perform the feature selection analyses. Heat maps and hierarchical clustering were constructed in the web-based chemometrics platform MetaboAnalyst 2.0 using the Pearson distance measure. For this specific analysis, protein spectral counts were previously z-score transformed.

#### Neighbor joining trees

In order to evaluate how similar the three classes were when considering their spectral counts distribution within the samples, the secretome dataset was analyzed using the neighbor joining (NJ) clustering method [38]. The phenetic trees were constructed from a Euclidean distance matrix using the VisPipeline software (http://vicg.icmc.usp.br/infovis2/Tools), developed at Instituto de Ciências Matemáticas e de Computação, Universidade de São Paulo, USP, São Carlos, Brazil. The silhouette coefficients [39] were also calculated for the 18 secretome dataset samples (both the raw data and their NJ clustering) using the VisPipeline software. The closest to 1 the silhouette coefficient (ranging from −1 to 1), the more efficient is data clusterization. Silhouette coefficients were also calculated after feature selection, in order to check for the coefficients improvement.

#### Identification of candidate biomarkers

The univariate Beta-binomial model was used to test the significance of protein differential abundances expressed in spectral counts in our label-free mass spectrometry-based proteomics dataset. The Beta-binomial model was constructed using a software package implemented in R according to Pham et al. [21].

In addition, protein spectral counts were submitted to other two different approaches: the semi-multivariate Nearest Shrunken Centroids (NSC) and the multivariate Support Vector Machine-Recursive Features Elimination (SVM-RFE). The NSC and the SVM-RFE models were also performed using software packages implemented in R according to Tibshirani et al. [40] and Guyon et al. [41], respectively. For both methods, a double cross-validation procedure was applied to define the optimal feature (protein) subsets (N) from the ranked proteins lists (independently ranked by each method). In the case of the SVM-RFE model, the optimal feature subset was the smallest set that provided the minimum mean classification error, whereas for the NSC model it was the subset that minimized the classification error and maximized the sum of true class probabilities [22]. The double cross-validation procedure was developed and implemented in R based on the work of Christin et al. [22]. Both feature selection methods had their performance assessed in terms of accuracy, sensitivity and specificity, using the caret package implemented in R [42]. The output final ranked lists of candidate biomarkers that resulted from each model (N defined by *p* < 0.05, in the case of the Beta-binomial model, or by double cross-validation, in the case of NSC and SVM-RFE models) were also compared with each other using the Jaccard similarity coefficient and a Venn diagram and considered for further analyses.

To compare our results to a classical statistical method, besides the methods described above, the univariate ANOVA test was also performed in our data using the ScaffoldQ+ software, with N defined by *p* < 0.05.

The same feature selection analyses using the three methods were performed for a published proteomics dataset of prostate cancer [23] in order to validate our proposed pipeline. The output final ranked lists of candidate biomarkers that resulted from each model were also compared by a Venn diagram.

#### Double cross-validation

The double cross-validation (DCV) is a type of statistical validation stricter than the cross-validation (CV), as in DCV a CV is performed within another CV. A CV error is an inappropriate estimate of the prediction error of the model, since this error is not based on an independent test set, as all data – both test and training samples – are used at once. Therefore, in order to avoid overly optimistic performance estimates, a “nested” CV scheme was performed in the DCV to estimate the prediction error, in which the parameter optimization is executed in an internal loop (inner loop) and the prediction error is estimated in an external loop (outer loop) on a completely independent set of samples [22, 43]. More details about how double cross-validation was used in NSC and SVM as well as the estimation of protein class are available in the Supplementary Material and Methods.

### Bioinformatics analyses

To explore the biological significance of the variables that greatly contributed to the characterization of each tumor class, protein-protein interaction networks were constructed using the Integrated Interactome System (IIS) software [44], developed at Laboratório Nacional de Biociências, CNPEM, Campinas, Brazil, for the candidate biomarkers identified by either the Beta-binomial, NSC and SVM-RFE models, and further estimated to be associated to the carcinoma or melanoma classes (Table E4). Enrichment analyses were performed in the networks using the IIS software for the curated pathways from the Kyoto Encyclopedia of Genes and Genomes (KEGG) [45]. Significantly enriched KEGG pathways (*p* ≤ 0.05) for proteins of carcinoma and melanoma secretomes were assigned as clusters in the networks and different colors and sizes were attributed to proteins proportionally to their fold change compared to the non-cancerous secretome class (−1.3 ≥ FC ≥ 1.3). Zero values were replaced by one in order to calculate the fold change. The resultant networks were visualized using the Cytoscape 2.8.2 software [46].

To evaluate whether the candidate biomarkers for melanoma and carcinoma were previously described related to cancer or to some biomarker application, a biomarker analysis was performed using the Ingenuity Systems Pathway software (IPA; Ingenuity Systems, Redwood City, CA). The Ingenuity biomarker filter module analysis was performed based on the following criteria: biofluids – “all”, disease – “cancer”, species – “human”, and biomarker application – “all”. Moreover, the Human Protein Atlas [47] was used to determine whether the retrieved candidates were previously indicated as cancer biomarkers.

### Immunoblotting

Proteins (5 μg) in the conditioned media from HaCaT, SCC-9, A431, A2058, SK-MEL-28 and HEK293 cell lines were separated under disulfide reducing conditions using SDS-polyacrylamide gel electrophoresis (SDS-PAGE) and transferred onto nitrocellulose membranes. The membranes were blocked in 5% dry milk in Tris-Tween buffered saline (TTBS). Membranes were then incubated overnight at 4°C with the following antibodies: anti-fibronectin (1:1000, Abcam), anti-tenascin-C (1:1000, Abcam), anti-GDF15 (1:1000, Abcam), anti-talin-1 (1:1000, Abcam), anti-EGFR (1:5000, Santa Cruz) and anti-CFB (1:1000, Abcam). Membranes were washed, incubated in horseradish peroxidase conjugated secondary antibodies and developed using enhanced chemiluminescence detection according to the manufacturer's instructions (Amersham Biosciences).

### Tissue array immunohistochemistry and statistical analysis

High density tissue microarrays were obtained from Biomax (OR601a and ME1004a). The presence of Complement Factor B (CFB) and Complement Component 3 (C3) was analyzed in 10 cancer-adjacent normal tissues and in 47 primary oral squamous cell carcinomas by immunohistochemistry using the streptavidin-biotin peroxidase complex (Dako). For tenascin-C and GDF15, 20 benign nevoid lesions (intradermal and compound nevus), 50 primary melanomas and 20 metastatic melanomas were subjected to immunohistochemical analysis with phosphatase alkaline/permanent red-based method (Dako). More details about the protein quantification of the tissue microarray staining intensity are available in the Supplementary Material and Methods.

### Small interfering RNA transfection

For silencing CFB gene, 3 × 10^5^ skin-derived epidermoid carcinoma (A431) cells were seeded in a six-well culture plate and transfected with 50 nM small interfering RNA (siRNA) duplex (sc-44510, Santa Cruz) and Lipofectamine 2000 according to the manufacturer's instructions (Invitrogen). Random stealth siRNA duplexes coding for nonfunctional RNAs served as control (sc-37007, Santa Cruz). After 72 h of incubation at 37°C and 5% CO_2_ atmosphere, transfection success was evaluated by real-time quantitative PCR and the cells have proceeded immediately for cell migration assay as described below.

### Real-time quantitative PCR

Skin-derived epidermoid carcinoma (A431) cells had their total RNA extracted by TRIzol reagent (Invitrogen Corporation), and 2 μg of total RNA were used for retro-transcription with a First-Strand cDNA Synthesis Kit (GE Healthcare). Real-time quantitative PCR for CFB was performed using a SYBR Green PCR Master Mix (Applied Biosystems), and dissociation curves were generated to confirm the specificity of the products. The threshold cycle (CT) values of the targeted gene were normalized relative to the glyceraldehyde-3-phosphate dehydrogenase gene expression, and relative expression ratios were calculated using the 2^−ΔΔ^ Ct method. Three independent experiments were performed in triplicates. The following PCR primers were used: CFB forward 5′-TCTCAG TCATTCGCCCTTCA-3′ and reverse 5′-CCTACGCTGACCTTGAT-3′; GAPDH forward 5′-GAAGGTGAAGGTCGGAGTCAAC-3′ and reverse 5′-CAGAGTTAAAA GCAGCCCCTGGT-3′.

### *In vitro* differentiation of macrophages derived from monocytes

Peripheral blood mononuclear cells (PBMCs) were collected from healthy volunteers through apheresis, performed in a Trima Accel System (Cobe BCT, Denver, CO, USA), at the Hospital Alemão Oswaldo Cruz, São Paulo, Brazil, after informed consent of donors. This procedure was approved by the Research Ethics Committee of the same institution. The procedure for differentiation of macrophages derived from monocytes is described in the Supplementary Material and Methods.

### Transwell migration assay

Untreated (mock), control siRNA-transfected (scramble) and CFB siRNA-transfected skin-derived epidermoid carcinoma (A431) cells (3 × 10^5^ cells) allowed to migrate for 16 h toward the lower chamber containing RPMI medium supplemented with 1% FBS. Two independent experiments were performed in triplicate.

For the co-culture assay, macrophage cells (7.5 × 10^4^ cells) were added in the upper chamber, and either mock, scrambled or CFB siRNA-transfected A431 cells (7.5 × 10^4^ cells) were added into the transwell plate lower chamber in 150 μl of serum-free RPMI-1640. At the end of the assay, the remaining cells at the top chamber were removed using a cotton swab, whereas the cells at the bottom of the insert filter were fixed with 10% formaldehyde for 10 min, washed with PBS and stained with 1% toluidine blue solution in 1% borax for 5 min. The dye was eluted in 1% SDS and absorbance was measured at 620 nm. Two independent experiments were performed in triplicate.

## SUPPLEMENTARY DATA, MATERIALS, METHODS, FIGURES AND TABLES




