# Supplementary material for: Integrative analysis to select cancer candidate biomarkers to targeted validation
Source: Oncotarget. 2015 Oct 30;6(41):43635–52. doi: 10.18632/oncotarget.6018 (PMC4791256; doi:10.18632/oncotarget.6018)
Supplement: Supplementary file 1 [file oncotarget-06-43635-s001.pdf]

SUPPLEMENTARY DATA

SUPPLEMENTARY MATERIALS AND METHODS

Mass spectrometric analysis

Label-free Discovery Proteomics: An aliquot containing 2.2 μg of proteins was analyzed on an ETD-enabled LTQ Orbitrap Velos Mass Spectrometer (Thermo Fisher Scientific) connected to a nanoflow liquid chromatography column (LC-MS/MS) by an EASY-nLC System (Proxeon Biosystem) through a Proxeon nanoelectrospray ion source. Peptides were separated by a 2–90% acetonitrile gradient in 0.1% formic acid using a pre-column EASY-Column (2 cm × id 100 μm, 5 μm particle size), and an analytical column PicoFrit Column (20 cm × ID75 μm, 5 μm particle size, New Objective), at a flow of 300 nl/min over 212 min. The nanoelectrospray voltage was set to 1.7 kV, and the source temperature was 275°C. All instrument methods for the LTQ Orbitrap Velos were set up in the data-dependent analysis (DDA) mode. The full scan MS spectra (m/z 300–2000) were acquired in the Orbitrap analyzer after accumulation to a target value of $1e^6$. The resolution in the Orbitrap was set to $r = 60{,}000$. The 20 most intense peptide ions with charge states ≥2 were sequentially isolated to a target value of 5,000 and fragmented in the linear ion trap by low-energy CID (normalized collision energy of 35%). The signal threshold for triggering an MS/MS event was set to 500 counts. Dynamic exclusion was enabled with an exclusion size list of 500, an exclusion duration of 60 s, and a repeat count of 1. An activation $q = 0.25$ and an activation time of 10 ms were used.

Label-free Targeted Proteomics: An aliquot containing 600 ng of digested proteins from saliva containing 5 fmol/μl of spiked synthetic angiotensin peptide was analyzed on an LTQ Orbitrap Velos mass spectrometer as previously described. Briefly, peptides were separated by a 2–90% acetonitrile gradient in 0.1% formic acid using an analytical column PicoFrit Column (20 cm × ID75 μm, 5 μm particle size, New Objective), at a flow of 300 nl/min over 80 min. The resolution in the Orbitrap was set to $r = 60{,}000$. The AGC target was $1.00e^5$ for SIM scans in the Orbitrap mass analyzer and $1.00e^4$ for MS/MS scans in the ion trap mass analyzer. The wide SIM windows were defined between 15 amu over the mass range of the selected C3 and CFB peptides. Targeted MS/MS was performed in the linear ion trap using global scheduled inclusion lists. The data were analyzed using the Xcalibur software (Thermo Fisher Scientific) to determine the extracted ion current peak area for MS1 and three transitions for each targeted peptide. Two technical replicates from each sample were performed. Each peptide was normalized by dividing the individual peptide (precursor area or the sum of three ion transitions/peptide) by the individual angiotensin reference peptides (precursor area or the sum of three ion transitions/peptide).

Data analysis

Label-free quantitation in data dependent analysis: Peak lists (msf) were generated from the raw data files using the Proteome Discoverer software version 1.3 (Thermo Fisher Scientific) with the Sequest search engine and searched against the Human International Protein Database (IPI) v. 3.86 (91,522 sequences; 36,630,302 residues), with the following parameters: carbamidomethylation as the fixed modification, oxidation of methionine as the variable modification, one trypsin missed cleavage and a tolerance of 10 ppm for precursor and 1 Da for fragment ions. All datasets were processed using the workflow feature in the Proteome Discoverer software, and the resulting search data were further analyzed in the software ScaffoldQ+v.3.3.1. The scoring parameters (Xcorr and Peptide Probability) in the ScaffoldQ+ software were set to obtain a false discovery rate (FDR) of less than 1%, using the number of total spectra output from the ScaffoldQ+ software. The peptide thresholds were: 60.0% minimum and Sequest: XCorr scores of greater than 1.8, 2.2, 2.5 and 3.5 for singly, doubly, triply and quadruply charged peptides, respectively, and the protein thresholds for identification were 90.0% minimum and 1 peptide minimum. A normalization criterion, the quantitative value, was applied to the spectral counts [38, 39].

Label-free targeted proteomics analysis

We selected two peptides for each protein based on the following criteria: uniqueness, high relative abundance, MS/MS spectral quality, experimental observation of proteomic data repositories (PeptideAtlas) and DDA analysis performed using LTQ Orbitrap Velos. The targeted proteomics were performed using selected ion monitoring (SIM) of each targeted peptide in high mass resolution for quantitation, followed by scheduled MS/MS for confirming targeted peptide sequences (Supplementary Table S15). The peak area of each targeted peptide was extracted using the Xcalibur software (Supplementary Table S16) and normalized to the angiotensin internal standard (Supplementary Table S17), spiked in all of the samples to a final concentration of 5 fmol/μl to correct run-to-run variations.

All mass spectrometric raw and msf files associated with this study are available for download via FTP from the PeptideAtlas data repository by accessing the following link: http://www.peptideatlas.org/PASS/PASS00388.

Double cross-validation

NSC (Nearest Shrunken Centroids)

The NSC double cross-validation was developed as follows:

1. Each inner loop (from a total of five) which is associated to an outer loop (from a total of six) calculates one model and one ranking using 90 threshold values. Many variables are discarded based on the threshold values and on the data set. A test data set associated to each inner loop is used to test each model and threshold combination. For each model the threshold that gives the minimum error with respect to the test data set and maximum probability score is recorded.

2. At the end of each outer loop, the maximum threshold among the five inner loops thresholds, that gives the minimum error and maximum probability overall is selected (resulting in the minimum N).

3. The thresholds for each outer loop are then applied to the training data set for model calculation and are challenged with the respective test data sets. The outer loop threshold is then selected by the minimum error, followed by the maximum probability and by the maximum threshold value.

4. The double cross-validation error is the average of the errors of the inner thresholds applied to their respective outer loops.

The NSC double cross-validation was repeated 100 times, and the final double cross-validation error was calculated as the closest value to the average of all 100 errors. N was then selected from this final double cross-validation error, followed by the maximum probability and by the maximum threshold value associated with the final error.

SVM-RFE (Support Vector Machine-Recursive Features Elimination)

In the case of SVM-RFE, the majority of calculated Ns in the inner loops were extremely small, ranging between one and four. Thus, when applying these Ns in the outer loops, models with only a few variables (approximately four) were constructed, and when using the test data set of the outer loops, errors ended up being extremely large (around 50%). Therefore, a different approach for the SVM-RFE double cross-validation was developed as follows:

1. Instead of using the minimum N from the inner loops of each outer loop, the mean error from the five inner loops is calculated for each value of N. Then, a minimum N that also minimizes e(N) is selected, where e(N) is the mean error of the inner loops when the first N proteins are considered.

2. The process is repeated for the six outer loops, again selecting the minimum N minimizing e(N). In general, the value of N presenting minimal error is selected over 30 training subsets.

3. Because the value of N is calculated by the average of the five inner loops, the ranking is also calculated based on such loops. For this calculation, the vectors W[i] of weights of each variable for each inner loop are multiplied. Thus, the ranking within an outer loop is the product vector W' = W[1]W[2]W[3]W[4]W[5].

4. This weight vector W' will define the ranking for each outer loop. This rank, with N selected from the corresponding inner loop, is used to construct the SVM-RFE model for a loop and to calculate its error.

5. The double cross-validation error is the average of the errors of the six outer loops.

The SVM-RFE double cross-validation was repeated 100 times, and the final double cross-validation error was calculated as the closest value to the average of all 100 errors. N was then selected as the value associated with this final double cross-validation error.

Estimation of protein classes

After the feature selection analyses, aiming to characterize the retrieved proteins as candidate biomarkers of each cell type, the secretome class in which each protein abundance changed the most among all classes was estimated. For that, a routine was developed and implemented in R, as further described. Proteins were associated to one of the three classes (melanoma, carcinoma or non-cancerous) using decision boundaries based on protein average spectral count values found in each class. After calculating the average values (hence, one value for each class, e.g., $x \le y \le z$), two boundaries were established as the mathematical intermediate point between the average values (B1 = $(x + y)/2$ and B2 = $(y + z)/2$). The average values were then associated to their closest boundary and classes were assigned index 1 if closest to the boundary with the lowest value, or 2 in the opposite case. In the example above, x would always be associated to B1 and z to B2; y attribution, however, would depend on how far x and z were from y. If y was associated to B1, both x and y classes were labeled with index 1; z class, otherwise, would score 2 and would be the class in which the corresponding protein abundance changed the most compared to the other two classes. If y was associated to B2, y and z would be labeled with index 2, whereas x would score 1 and would be the class

in which the corresponding protein abundance is the most altered (Supplementary Table S3, "Class boundary index" column). These indexes were used to classify proteins in the dataset, considering that proteins are candidate biomarkers of the secretome classes where the average abundance values are the most altered compared to the other classes.

Tissue array immunohistochemistry and statistical analysis

Protein quantification was assessed with the aid of Aperio Scanscope CS® Slide Scanner and the Pixel Count V9 algorithm software (Aperio Technologies, Vista, CA; USA). By using specific input parameters, the percentage of cytoplasm positivity was calculated and classified as weak, moderate and strong, according to its staining intensity. Each category received an intensity score, 1 to weak, 2 to moderate and 3 to strong staining. The final score of each tissue sample was calculated as the sum of the percentage of each category multiplied by their respective intensity scores as the following formula: Score = (%weak × 1) + (%moderate × 2) + (%strong × 3). Mann Whitney U and one-way ANOVA (p-value < 0.05) were used to compare protein quantities between groups.

In vitro differentiation of macrophages derived from monocytes

For the enrichment of mononuclear cells, the product of apheresis was submitted to a separation with Ficoll-Paque (GE Healthcare Bio-Sciences AB, Uppsala, Sweden) for 30 min at 900 × g at 18°C. Mononuclear cells were collected and centrifuged at 600 × g for 10 min at 18°C with RPMI-1640 medium (Gibco, Grand Island, NY, EUA) and washed twice with RPMI-1640 medium at 300 × g and 200 × g, respectively. The mononuclear cells were then resuspended in R10 supplemented with 1% of Antibiotic-Antimycotic (Gibco, Grand Island, NY, EUA), seeded in a 24-well plate (3×10^6 cells/ml suspension) and incubated at 37°C and 5% CO_2 atmosphere for 2 h. After this period, non-adherent cells were removed and cell medium was supplemented with 50 ng/ml GM-CSF (PeproTech, Rocky Hill, NJ, USA). These cells were maintained in culture for seven days for differentiation of monocytes into macrophages.

Supporting information available

This material is available free of charge via the Internet at http://pubs.acs.org.

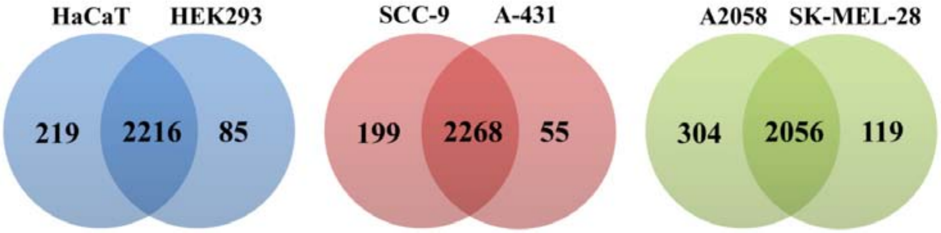

Supplementary Figure S1: Venn diagrams illustrate the number of exclusive or shared proteins by the cell lines of each class.

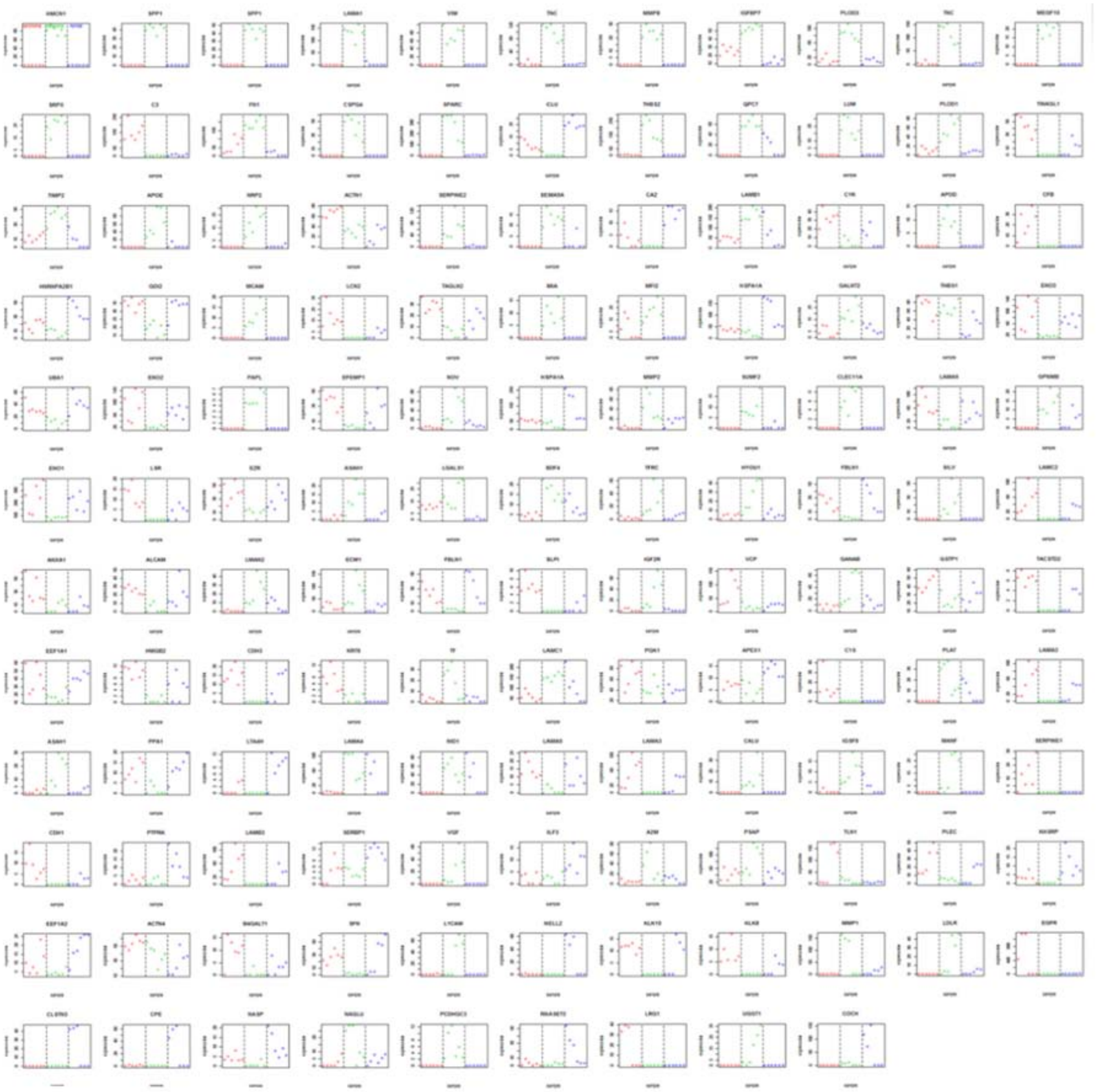

Supplementary Figure S2: Plots showing the spectral counting distribution in the melanoma, carcinoma and non-cancerous cells for the 130 candidate biomarkers retrieved from the NSC model.

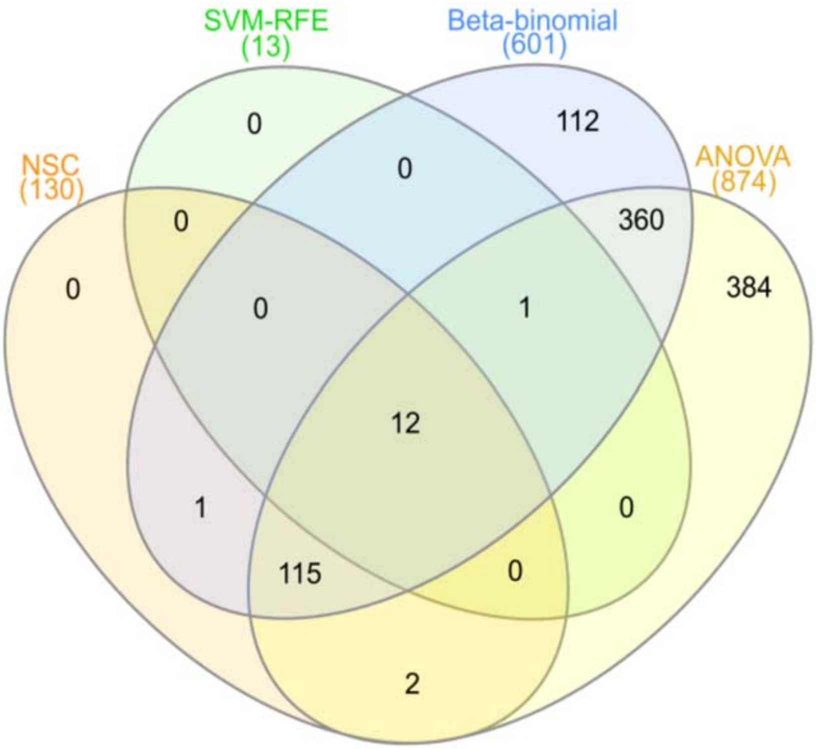

Supplementary Figure S3: Immunoblotting (IB) corroborates mass spectrometry (MS) findings. The selected proteins analyzed by IB presented results comparable to the quantification by MS (depicted as z-score-transformed heat maps). Conditioned media from HaCaT, HEK293, A-431, SCC-9, A2058 and SK-MEL-28 cells were concentrated, and 5 µg of total protein for each lane was loaded onto SDS-PAGE gels. Then, proteins were transferred onto nitrocellulose membrane and probed with the indicated protein antibodies.

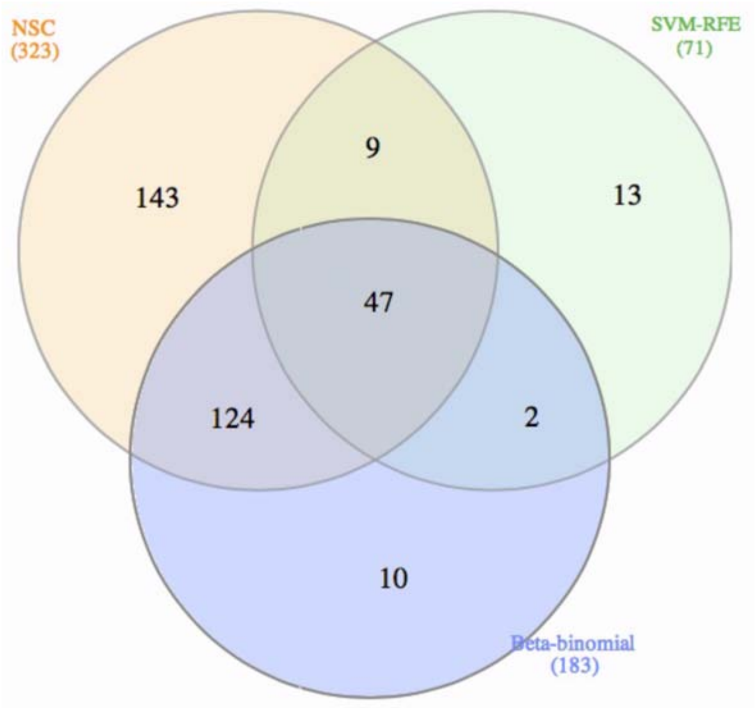

Supplementary Figure S4: Comparison of the three feature selection methods (Beta-binomial, SVM-RFE and NSC) applied to a published dataset of OC (organ-confined) and EC (extracapsullar) prostate cancer cells (Kim et al., 2012) to identify candidate biomarkers between the two tumor classes. The Venn diagram shows the intersections among the optimal feature subsets (N) retrieved by the three methods.

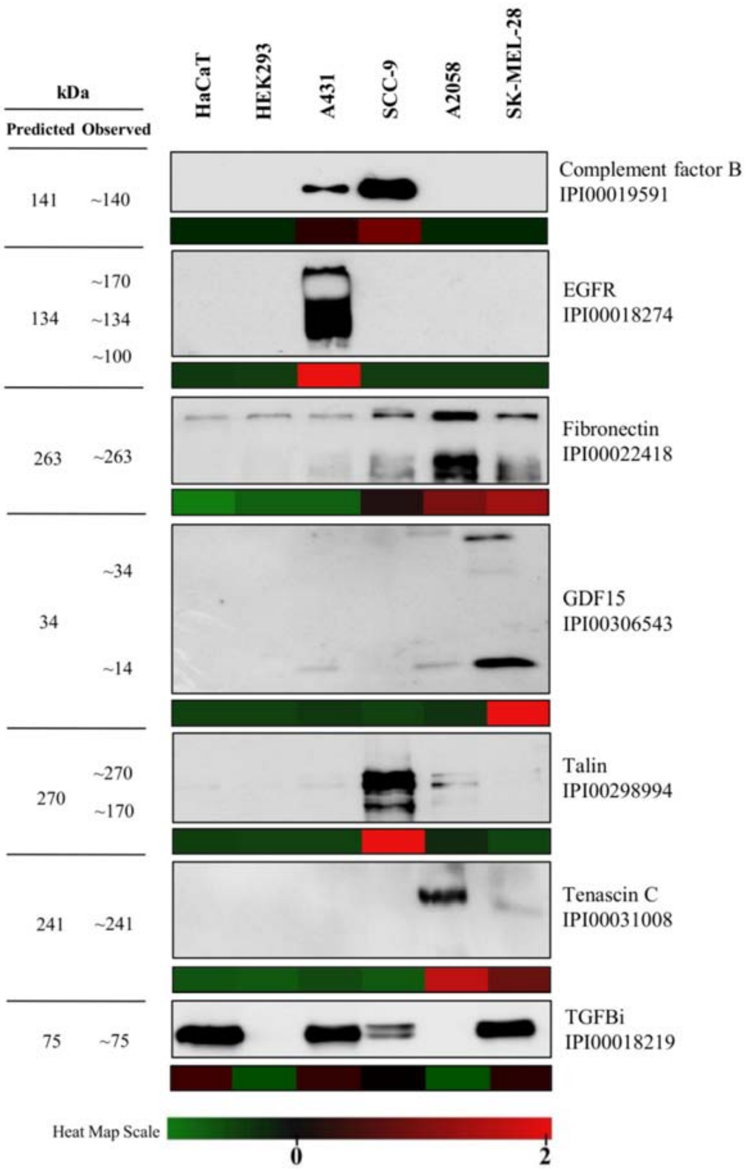

Supplementary Figure S5: Venn diagram showing the intersections among the optimal feature subsets (N) retrieved by four feature selection methods: the three included in the proposed discovery-to-target pipeline and the classical ANOVA statistical test.

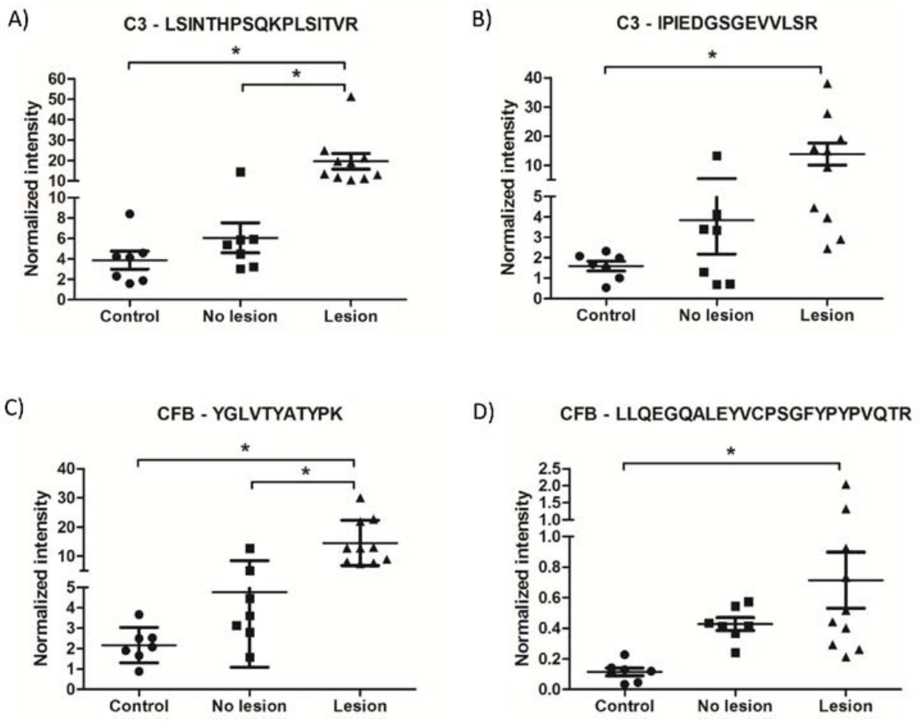

Supplementary Figure S6: CFB and C3 peptides showed higher normalized intensity (sum of three transitions) in OSCC saliva samples than in healthy saliva samples. PseudoSRM approach for peptides of C3 (precursor m/z 631.05, +3; 735.89, +2) and CFB (precursor m/z 638.33, +2; 939.13, +3) normalized to 5 fmol of angiotensin (m/z 432.89, +3) as an internal reference peptide. These data represent two technical replicates of saliva samples from healthy patients (n = 7), saliva samples from patients who undergone surgical resection of OSCC (named no lesion, n = 7) and saliva samples from patients with active OSCC lesion without any treatment (named lesion, n = 10) (ANOVA followed by Tukey's test). The data were normalized by dividing the targeted angiotensin peptide peak area (sum of three transitions) by the peak area of each C3 and CFB peptide (sum of three transitions).

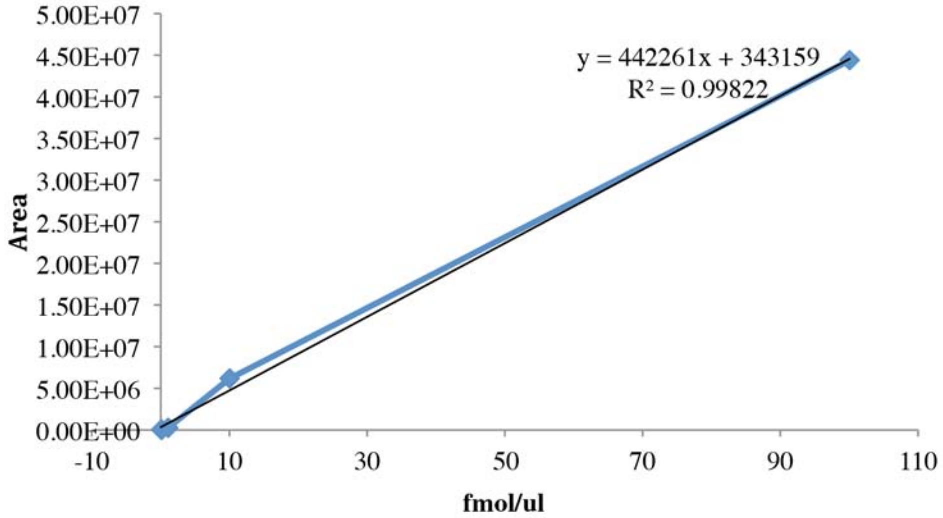

Supplementary Figure S7: Standard curve for angiotensin peptide spiked at different concentrations into the HEK293 digest. Angiotensin peptide was spiked in 500 ng of the HEK total extract protein digest at increasing concentrations (0.01; 0.1; 1; 10 and 100 fmol/µl). Angiotensin peptide was targeted employing single ion monitoring of precursor m/z 432.89, +3. The peptide identity was confirmed by scheduled CID MS/MS. Each sample was run in triplicate.

Supplementary Table S1: List of proteins identified by mass spectrometry in the conditioned media of HaCaT, HEK293, A-431, SCC-9, A2058 and SK-MEL-28 cell lines.

Supplementary Table S2: List of all identified peptide sequences by mass spectrometry in the conditioned media of HaCaT, HEK, A431, SCC-9, A2058 and SK-MEL-28 cell lines.

Supplementary Table S3: Table S3 Correlation analysis (R-squared) of pair-samples using the whole proteome dataset

Supplementary Table S4: Complete ranked protein lists from carcinoma, melanoma and normal (non-cancerous) cells resultant from the three feature selection methods: Beta-binomial, NSC and SVM-RFE. The ANOVA test was also performed and the results are depicted in the table but was not included in the pipeline.

Supplementary Table S5: Putative carcinoma and melanoma biomarkers identified by three different methods: Beta-binomial, NSC and SVM-RFE. Dots are used to depict the corresponding methods.

Supplementary Table S6: Ranked protein list from OC (Organ-confined) and EC (extracapsullar) prostate cancer cells resultant from Beta-binomial analysis. (Kim et al., 2012, Molecular and Cellular Proteomics).

Supplementary Table S7: Statistical metrics calculated to assess the performance of SVM-RFE and NSC models.

Supplementary Table S8: Ranked protein list from OC (Organ-confined) and EC (extracapsullar) prostate cancer cells resultant from NSC analysis. (Kim et al., 2012, Molecular and Cellular Proteomics)

Supplementary Table S9: Ranked protein list from OC (Organ-confined) and EC (extracapsullar) prostate cancer cells resultant from SVM-RFE analysis. (Kim et al., 2012, Molecular and Cellular Proteomics).

Supplementary Table S10: Protein lists retrieved from a Venn diagram analysis after OC (Organ-confined) and EC (extracapsullar) prostate cancer cells selection feature analyses. Each column shows the list corresponding to the intersection or exclusive set of the Venn diagram. A number next to the set description correspond to the number of proteins in each set. Highlighted proteins in yellow (Gene_Uniprot) correspond to the six candidate biomarkers verified by different methods in the original paper by Kim et al., 2012, Molecular and Cellular Proteomics.

Supplementary Table S11: Biomarker filter analysis using Ingenuity and Human Protein Atlas applied to the list of putative prostate cancer biomarkers. (Kim et al., 2012, Molecular and Cellular Proteomics) identified by the intersection of three different methods: Beta-binomial, NSC and SVM-RFE.

Supplementary Table S12: Enriched KEGG pathways for each protein in the carcinoma network. Proteins for which no enriched terms were assigned are not shown. Only enriched KEGG pathways with $p \leq 0.05$ were considered in the network analyses. Proteins were assigned as down-, up- or non-regulated protein according to their carcinoma class mean spectral counts vs. non-cancerous class mean spectral counts fold change ($-1.3 \geq FC \geq 1.3$).

Supplementary Table S13: Enriched KEGG pathways for each protein in the melanoma network. Proteins for which no enriched terms were assigned are not shown. Only enriched KEGG pathways with $p \leq 0.05$ were considered in the network analyses. Proteins were assigned as down-, up- or non-regulated according to their melanoma class mean spectral counts vs. non-cancerous class mean spectral counts fold change ($-1.3 \geq FC \geq 1.3$).

Supplementary Table S14: Clinicopathological variables of the OSCC patients and healthy subjects.

Supplementary Table S15: Angiotensin and spiked-in C3 and CFB peptides and transitions selected for LC-pseudoSRM.

Supplementary Table S16: Extracted ion current peak area from all MS1 and the three transitions for each peptide using Xcalibur software.

Supplementary Table S17: Normalized intensity of precursor and transitions and CV% between the replicates.

Supplementary Table S18: Standard curve of internal reference angiotensin peptide spiked in a 500 ng HEK293 digest.
